# Supplementary material for: Need for split: integrative taxonomy reveals unnoticed diversity in the subaquatic species of Pseudohygrohypnum (Pylaisiaceae, Bryophyta)
Source: PeerJ. 2022 Apr 26;10:e13260. doi: 10.7717/peerj.13260 (PMC9053303; doi:10.7717/peerj.13260)
Supplement: Supplemental Information 2 [file peerj-10-13260-s002.docx]

Primer Reference Sequence (5′–3′) region

trnK1Hyp-F newly designed here ACTCAATGGTAGAGTACTCGGC trnK–psbA

trnK1Hyp-R newly designed here TCTTGTATMCGTCGACGAAGAATT trnK–psbA

trnK2Hyp-F newly designed here AGAAGCATAGCAGAAATTTTGYRCGA trnK–psbA

trnK2Hyp-R newly designed here TGTTTTAGCACAAGAAAATCGAAGT trnK–psbA

trnK3Hyp-F newly designed here TTCTTCGTCGACGKATACAAGA trnK–psbA

trnK3Hyp-R newly designed here CTCTTCCCAAACCGTGCTTG trnK–psbA

trnK4Hyp-F newly designed here KCGATTTTCTTGTGCTAARACATT trnK–psbA

trnK4Hyp-R newly designed here RAAGCGACCCCATARGCTTG trnK–psbA

rbcL-Bryo-2F newly designed here ATGTCTTCGTGGTGGACTTGA rbcL

rbcL-Bryo-2R newly designed here CCCTTGACTTYATGGTTCGGAA rbcL
