# Supplementary material for: Need for split: integrative taxonomy reveals unnoticed diversity in the subaquatic species of Pseudohygrohypnum (Pylaisiaceae, Bryophyta)
Source: PeerJ. 2022 Apr 26;10:e13260. doi: 10.7717/peerj.13260 (PMC9053303; doi:10.7717/peerj.13260)
Supplement: Supplemental Information 3 [file peerj-10-13260-s003.docx]

Table 3. Fossil records used for calibration.

| Taxon | Period | Source | Mean | St. Dev. |
| --- | --- | --- | --- | --- |
| Homalia | Early–middle Miocene | Dominican amber | 16.5 Ма | 1.5 |
| Thuidium | Early–middle Miocene | Dominican amber | 16.5 Ма | 1.5 |
| Brachythecium | Eocene | Baltic amber | 33.5 Ма | 0.5 |
| Boulaya | Eocene | Baltic amber | 33.5 Ма | 0.5 |
| Campylium | Eocene | Baltic amber | 33.5 Ма | 0.5 |
