# Supplementary material for: Need for split: integrative taxonomy reveals unnoticed diversity in the subaquatic species of Pseudohygrohypnum (Pylaisiaceae, Bryophyta)
Source: PeerJ. 2022 Apr 26;10:e13260. doi: 10.7717/peerj.13260 (PMC9053303; doi:10.7717/peerj.13260)
Supplement: Supplemental Information 4 [file peerj-10-13260-s004.docx]

| Variable | Name | Explanation | Unit | Source |
| --- | --- | --- | --- | --- |
| AI | Global Aridity — Annual (The Global Land Aridity Index) | the moisture availability as a ratio of mean annual precipitation to mean annual potential evapotranspiration | - | (CGIAR-CSI) Zomer et al. 2008 |
| Bio 1 | mean annual air temperature | mean annual daily mean air temperatures averaged over 1 year | °C/10 | (CHELSA data) Karger et al. 2017, 2018 |
| Bio 2 | mean diurnal air temperature range | mean diurnal range of temperatures averaged over 1 year | °C/10 | (CHELSA data) Karger et al. 2017, 2018 |
| Bio7 | annual range of air temperature | The difference between the Maximum Temperature of Warmest month and the Minimum Temperature of Coldest month | °C/10 | (CHELSA data) Karger et al. 2017, 2018 |
| Bio12 | annual precipitation amount | Accumulated precipitation amount over 1 year | kg m-2 | (CHELSA data) Karger et al. 2017, 2018 |
| Bio15 | precipitation seasonality | The Coefficient of Variation is the standard deviation of the monthly precipitation estimates expressed as a percentage of the mean of those estimates (i.e. the annual mean) | kg m-2 | (CHELSA data) Karger et al. 2017, 2018 |
| Bio17 | mean precipitation amount of the driest quarter | The driest quarter of the year is determined (to the nearest month) | kg m-2 | (CHELSA data) Karger et al. 2017, 2018 |
| Bio18 | mean monthyl precipitation amount of the warmest quarter | The warmest quarter of the year is determined (to the nearest month) | kg m-2 | (CHELSA data) Karger et al. 2017, 2018 |
| Bio19 | mean monthly precipitation amount of the warmest quarter | The coldest quarter of the year is determined (to the nearest month) | kg m-2 | (CHELSA data) Karger et al. 2017, 2018 |
