# Supplementary figures and images for: Need for split: integrative taxonomy reveals unnoticed diversity in the subaquatic species of Pseudohygrohypnum (Pylaisiaceae, Bryophyta)
Source: PeerJ. 2022 Apr 26;10:e13260. doi: 10.7717/peerj.13260 (PMC9053303; doi:10.7717/peerj.13260)

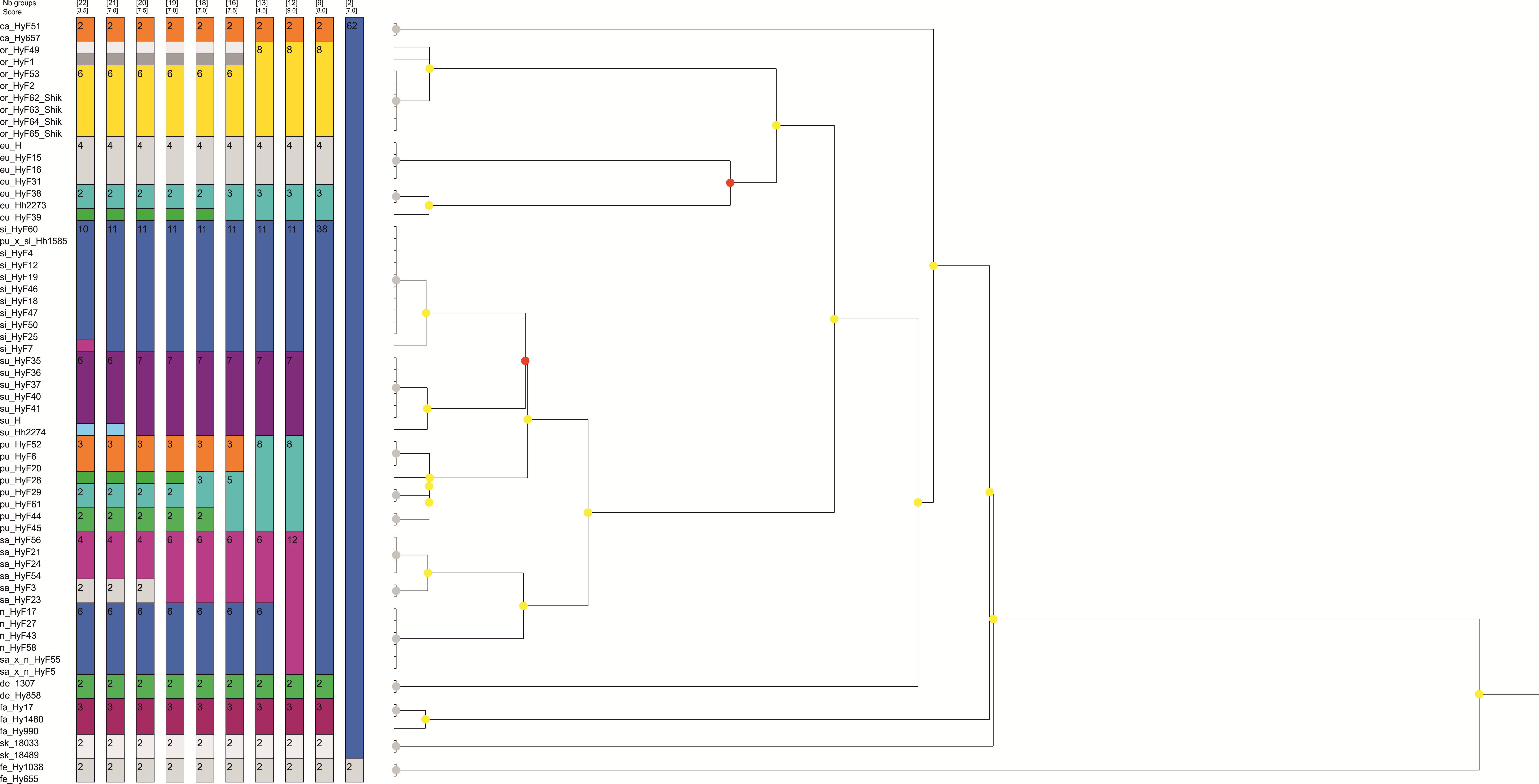

Supplement: Supplemental Information 8 [file peerj-10-13260-s008.png]

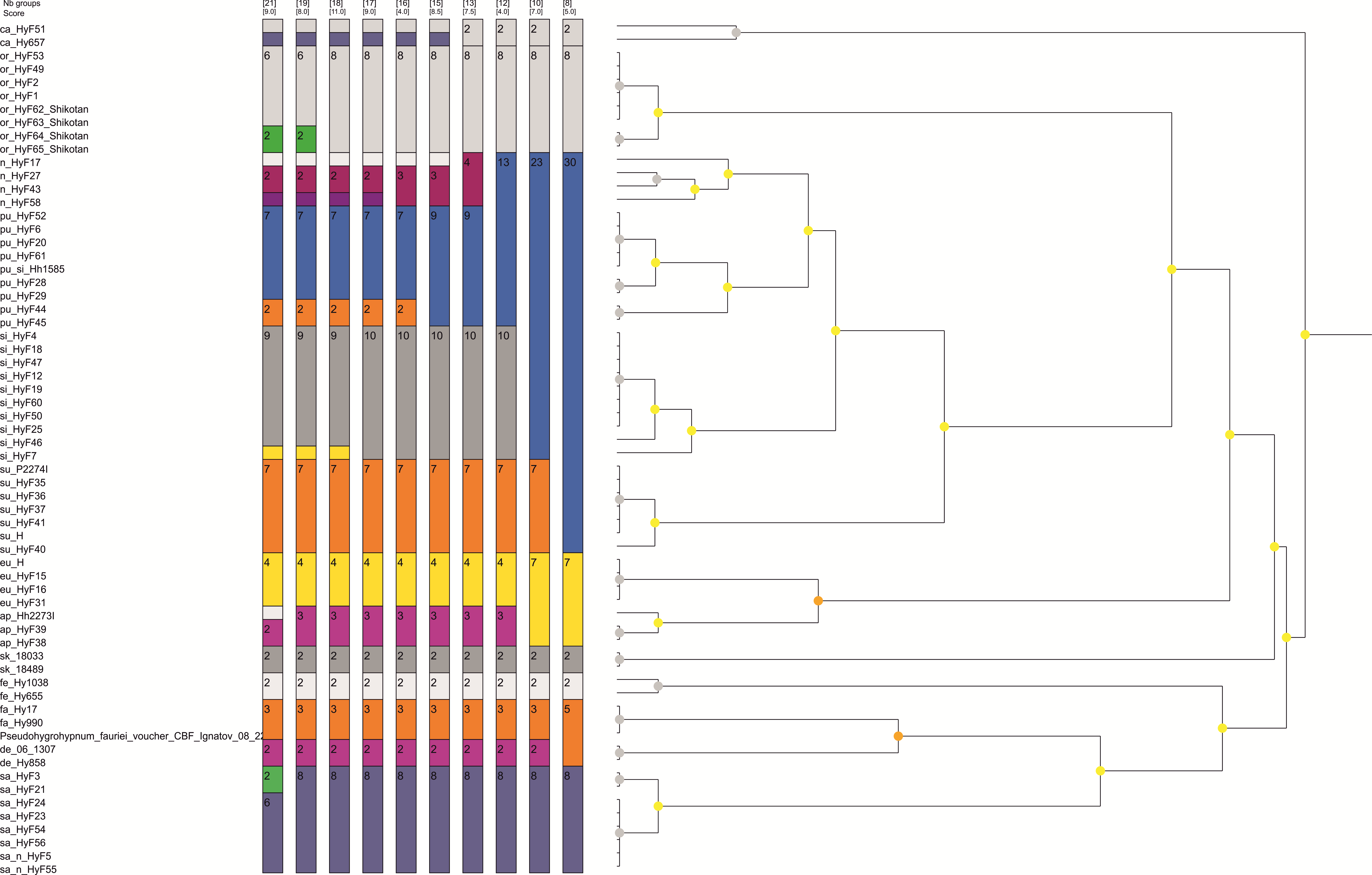

Supplement: Supplemental Information 9 [file peerj-10-13260-s009.png]
